# Protective effect of the methanol extract from *Cryptotaenia japonica* Hassk. against lipopolysaccharide-induced inflammation in vitro and in vivo

**DOI:** 10.1186/1472-6882-12-199

**Published:** 2012-10-30

**Authors:** Hee Kang, Tae-Sun Bang, Ji-Won Lee, Jae-Hwan Lew, Seok Hyun Eom, Kyungjin Lee, Ho-Young Choi

**Affiliations:** 1 Graduate School of East–west Medical Science, Kyung Hee University, Yongin-si, Gyeonggi-do, 446-701, South Korea; 2 Department of Herbology, College of Korean Medicine and Institute of Korean Medicine, Kyung Hee University, Seoul, 130-701, South Korea; 3 Department of Horticultural Biotechnology, College of Life Sciences, Kyung Hee University, Yongin-si, Gyeonggi-do, 446-701, South Korea

**Keywords:** *Cryptotaenia japonica* Hassk., Inflammation, Macrophages, iNOS, Cytokines, Signaling

## Abstract

**Background:**

In folk medicine, the aerial part of *Crytotaenia japonica* Hassk. (CJ), is applied for treatment of the common cold, cough, urinary problems, pneumonia, and skin rashes. In this paper, the in vitro and in vivo anti-inflammatory activity of CJ methanol extract was tested using lipopolysaccharide (LPS)-induced inflammatory models.

**Methods:**

We measured nitric oxide (NO), inducible NO synthase (iNOS), and inflammatory cytokine levels from LPS-stimulated mouse peritoneal macrophages. Also, several cellular signaling molecules which regulate the expressions of these inflammatory markers were examined. Finally, we tested whether oral administration of CJ methanol extract might affect the serum cytokine levels in LPS-injected mice.

**Results:**

CJ methanol extract reduced NO release via iNOS protein inhibition. The extract was also shown to decrease the secretions of tumor necrosis factor (TNF)-α, interleukin (IL)-6, and IL-12. Analysis of signaling molecules showed that CJ inhibited the phosphorylation of STAT1, p38, JNK and ERK1/2 as well as IκBα degradation. Finally, CJ decreased the serum levels of TNF-α and IL-6 in LPS-injected mice.

**Conclusions:**

Our results demonstrated the anti-inflammatory activity of CJ methanol extract and its possible underlying mechanisms that involve modulation of IκBα, MAPK, and STAT1 activities.

## Background

*Cryptotaenia japonica* Hassk. (CJ) belongs to the Apiaceae family and is a perennial plant distributed in Asia and North America. The aerial part of the plant is used both as a vegetable and a medicinal herb. In folk medicine, CJ is applied for treatment of the common cold, cough, urinary problems, pneumonia, and skin rashes.

Macrophages are professional phagocytes that reside in tissues throughout the body to remove cellular debris and effete cells generated under physiologic conditions [1]. Macrophages also constitute the major cellular components of the inflammatory response. Pathogenic microbes, their byproducts, and host-derived cytokines or other secreted products can stimulate macrophages. Whether the source of insult comes from within or outside the body, the receptors and subsequent signaling molecules employed are similar, resulting in the production of lipid mediators and inflammatory cytokines. However, these responses must be strictly controlled as they may damage healthy tissue and lead to chronic inflammatory disorders such as autoimmune disease, degenerative disease, and cancer [2].

Signals derived from pathogens or host cells, such as pathogen-associated molecular patterns (PMAP), danger-associated molecular patterns (DAMP) and interferon (IFN)-γ, can activate macrophages [3,4]. PAMP and DAMP are recognized by various pattern recognition receptors and ultimately cause the activations of mitogen-activated protein kinase (MAPK) and NF-κB signaling pathways, which result in the expressions of many inflammatory genes including inducible nitric oxide synthase (iNOS), tumor necrosis factor (TNF)-α and interleukin (IL)-6 and IL-12. IFN-γ, once known as macrophage activation factor, is produced by natural killer (NK) cells early in the immune response and later by type I T helper (Th1) cells. Binding of IFN-γ to its receptor causes the activations of JAK1,2-STAT1, which enhance the expressions of IFN-γ-regulated genes including those required for antigen processing and presentation, antiviral state, and microbicidal functions in macrophages [5].

Despite the long-lasting use of CJ in folk medicine, scientific evidence for its effectiveness is lacking. A recent study showed that the seed essential oils of CJ have antioxidant and hypolipidemic effects [6]. In this paper, we examined the protective effect of CJ using an lipopolysaccharide (LPS)-induced inflammation model in vitro and in vivo. We also investigated whether this plant modulates cellular signaling molecules which regulate the expressions of inflammatory markers.

## Results

### Identification of chemical constituents in the methanol extract of the aerial part of *Cryptotaenia japonica*

We performed gas chromatography/mass spectrometry in order to characterize the chemical constituents in the methanol extract of the aerial part of *Cryptotaenia japonica* (CJ). The identification of constituents was based on software, TurboMass using NIST library. Total components were listed in Table 1.

**Table 1:** **Identification of chemical constituents in the methanol extract of the aerial part of *Cryptotaenia japonica *by GC/MS analysis**

**Peaks**	**RT**^**z**^	**Constituents**
1	1.959	1,4,7-Trioxa-10-azacyclododecane
2	8.579	Catechol
3	8.867	Benzofuran, 2,3-dihydro-
4	9.385	Hydroquinone
5	11.748	N,N’-Ethylenebis(2-[2-hydroxyphenyl]glycine
6	11.923	Methyl 4-O-acetyl-2,3,6-tri-O-ethyl-alpha-d-galactopyrasnoside
7	12.024	(+)-alpha-Panasinsen
8	12.658	Fumaric acid, 2-ethylhexyl undecyl ester
9	14.394	3,7,11,15-Tetramethy-2-hexadecen-l-ol
10	15.012	Hexadecanoic acid, methyl ester
11	15.223	Pentadecanoic acid
12	15.800	Falcarinol
13	16.133	Methyl 7,12-octadecadienoate
14	16.174	7,10,13-Eicosatrienoic acid, methyl ester
15	16.231	Phytol
16	16.784	Card-20(22)-enolide,3,5,14,19-tetrahydroxyl-,

^Z^ indicates retention time. Chemical constituents were identified by referring to the computer mass libraries.

### Effects of CJ methanol extract on LPS-induced nitric oxide (NO) and inducible NO synthase

**Figure 1:**
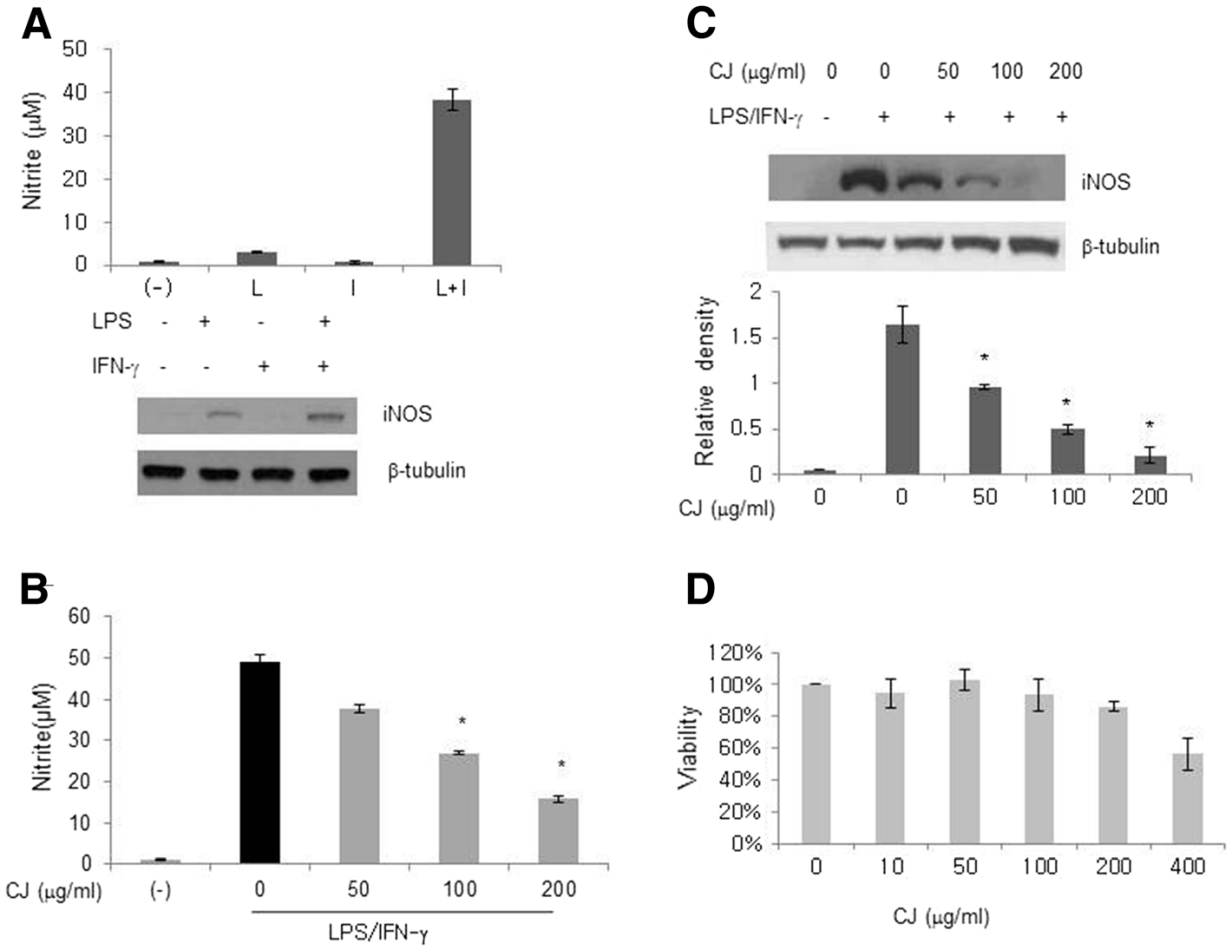
**Effect of the aerial part of *Cryptotaenia japonica *Hassk. (CJ) methanol extract on LPS-induced NO and iNOS.A**. Peritoneal macrophages were primed with IFN-γ (0.5 ng/ml) for 2 h and then stimulated with LPS (100 ng/ml) for 18 h. NO in the medium was detected by the Griess reaction. The expression of iNOS protein was analyzed by Western blotting. β-tubulin was loaded as an internal control. **B**, **C**: IFN-γ-sensitized cells were stimulated with LPS in the presence of the CJ methanol extract for 18 h. **D**: Cells were cultured with CJ methanol extract for 24 h and cell viability was determined using the MTS assay. Viability was presented as percent of control cells (0 μg/ml). Data represent mean ± SD of two to four independent assays. * denotes significant difference ( *P* < 0.05) from cells treated with LPS alone.

In an attempt to examine the anti-inflammatory effect of CJ methanol extract, we first measured the levels of nitric oxide (NO) produced by LPS-stimulated macrophages. In our in vitro system, pretreatment with IFN-γ increased the NO release in LPS-stimulated mouse peritoneal macrophages (Figure 1A). Therefore, peritoneal macrophages were primed with IFN-γ before the addition of LPS and CJ methanol extract for 18 h. Since NO is unstable and rapidly metabolizes to nitrate and nitrite, nitrite levels were used as an indicator of NO production. Treatment with CJ methanol extract for 18 h decreased NO in a concentration-dependent manner (Figure 1B). In the inflammatory condition, production of NO is mainly controlled by an enzyme called iNOS. Many inflammatory mediators including cytokines regulate the induction of iNOS [7]. As shown in Figure 1C, the detectable iNOS protein was observed in cells treated with LPS plus IFN-γ. Our results showed that concentration-dependent reduction of the iNOS protein by CJ methanol extract was much more potent than that of NO. Because treatment with CJ methanol extract was not cytotoxic up to 200 μg/ml (Figure 1D), the decreased iNOS synthesis was not likely to be due to a reduction in cell number.

### Effects of CJ methanol extract on LPS-induced inflammatory cytokines

**Figure 2:**
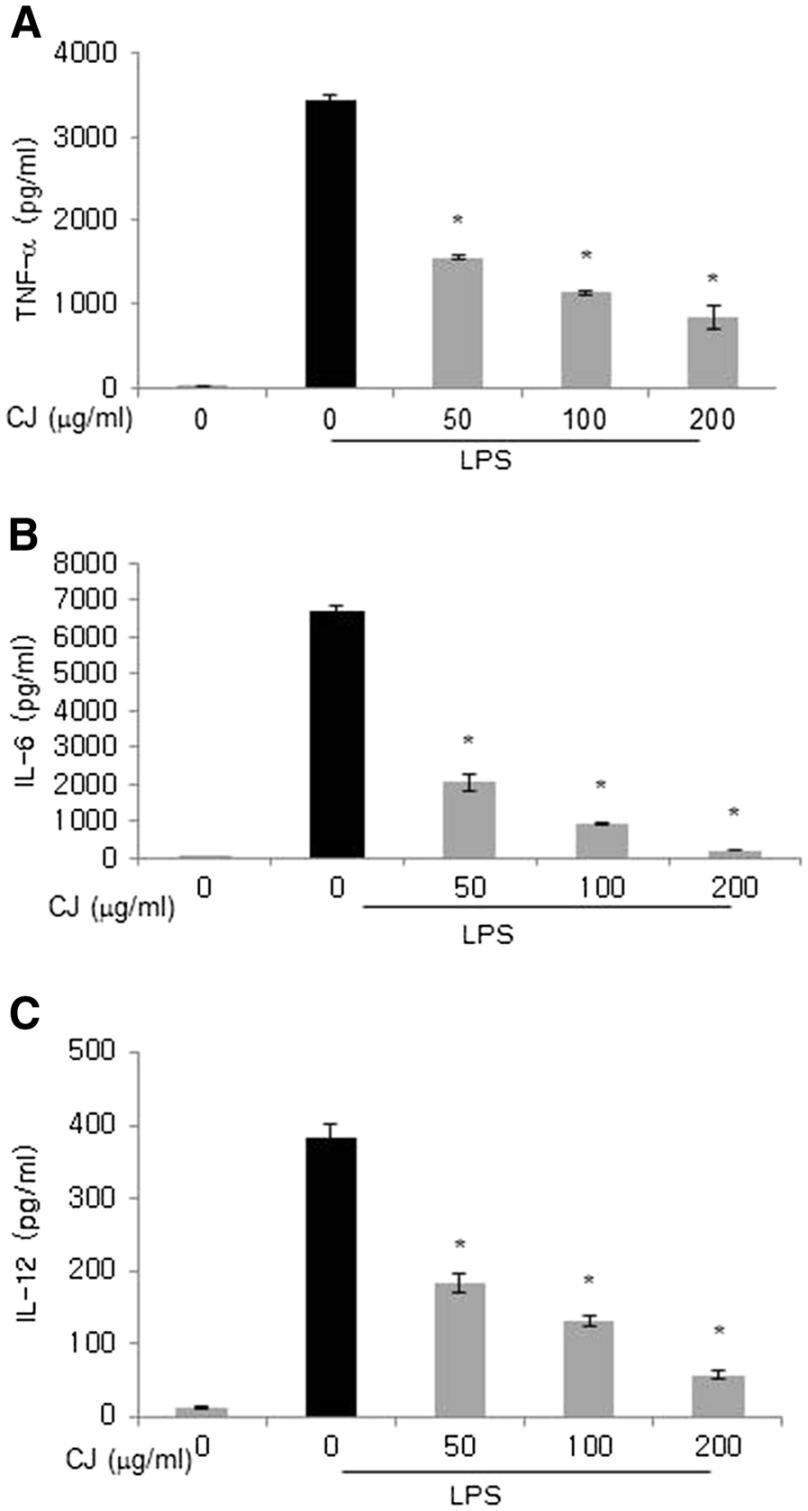
**Effect of CJ methanol extract on TNF-α, IL-6 and IL-12 in LPS-stimulated macrophages.** Peritoneal macrophages were stimulated with LPS (100 ng/ml) in the presence of CJ methanol extract for 18 h. Cytokine production in the culture medium was measured by ELISA. Data represent mean ± SD of four independent assays. * denotes significant difference ( *P* < 0.05) from cells treated with LPS alone.

Activated macrophages initiate the inflammatory response by secreting TNF-α, IL-6, and IL-12. We examined whether CJ methanol extract might influence the levels of these inflammatory cytokines in response to LPS using ELISA. As shown in Figures 2A-C, CJ methanol extract inhibited the release of the cytokines in a concentration-dependent manner.

### Effects of CJ methanol extract on IκBα degradation

**Figure 3:**
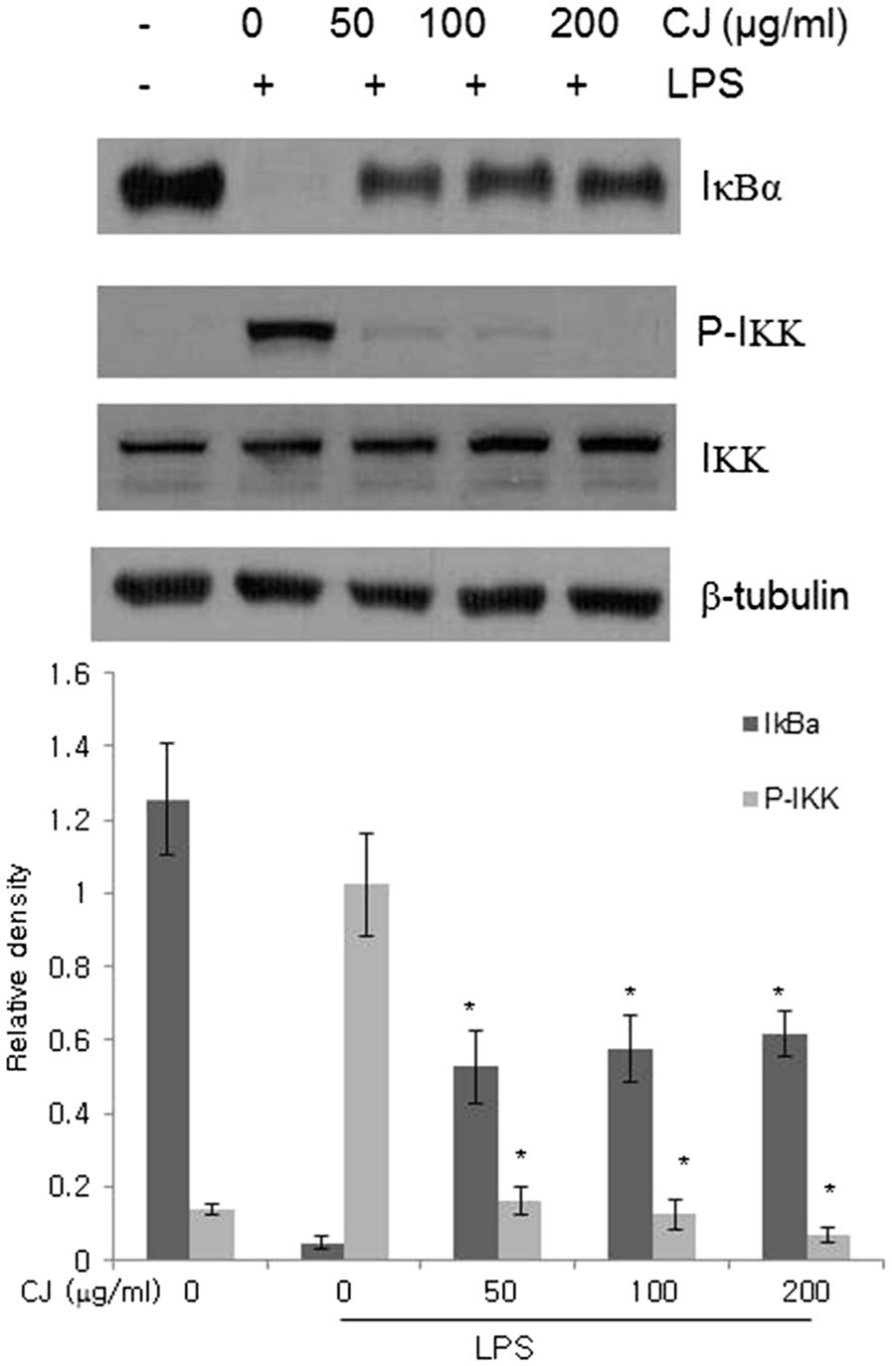
**Effect of CJ methanol extract on LPS-induced IκBα degradation and IKK activation.** Peritoneal macrophages were pretreated with CJ methanol extract 1 h followed by LPS stimulation for 15 min. Total protein was extracted and assessed by Western blotting. One of four experiments is shown. Data represent mean ± SD of four independent assays. * denotes significant difference ( *P* < 0.05) from cells treated with LPS alone.

IκBα is a key regulator of NF-κB proteins. In its inactive form, NF-κB is sequestered by IκBα in the cytosol; however, LPS causes the IκBα kinase (IKK) to catalyze the phosphorylation of IκBα, which results in the degradation of IκBα and the translocation of NF-κB to the nucleus [8]. At 15 min after LPS stimulation, IKK phosphorylation and IκBα degradation were observed in the control cells (Figure 3). Treatment with CJ methanol extract inhibited IκBα degradation and IKK activation. Noticeably, such reductions assayed at 50 – 200 μg/ml were concentration-independent. Based on these results, the inhibitory effect of CJ methanol extract may occur upstream of IKK activation in the NF-κB pathway.

### Effects of CJ methanol extract on MAPK signaling

**Figure 4:**
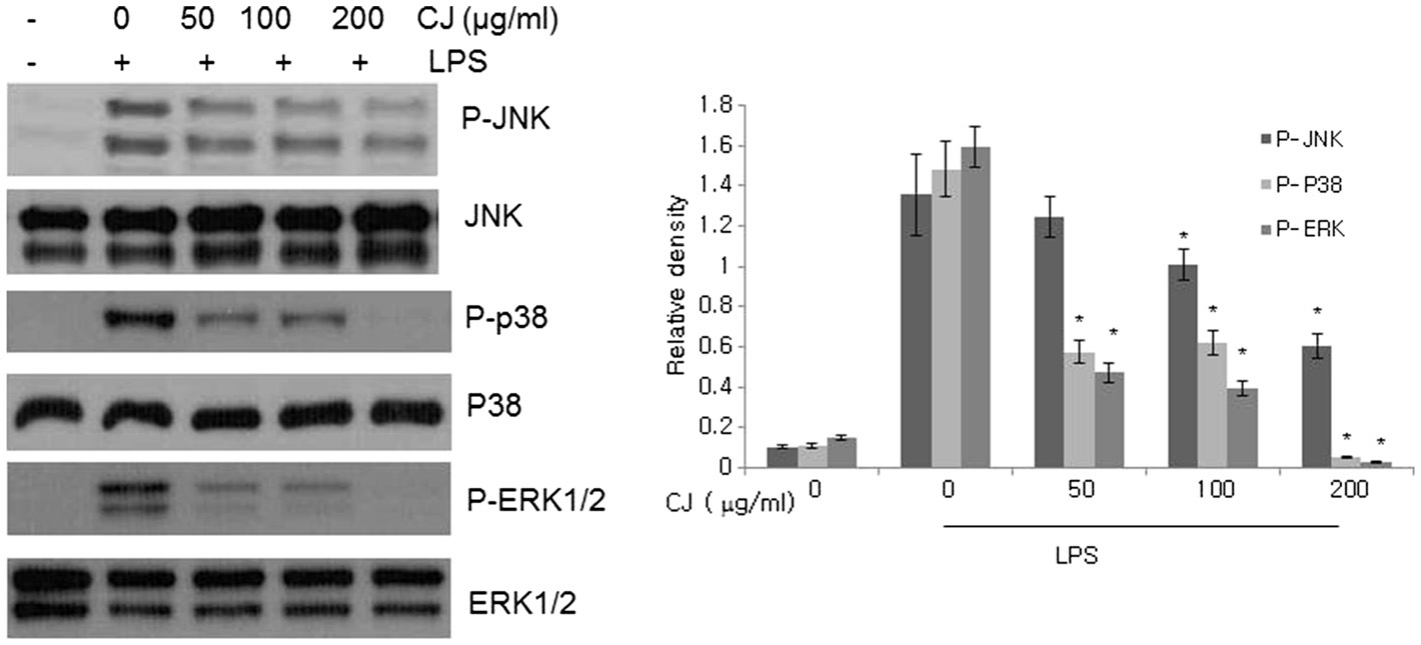
**Effect of CJ methanol extract on LPS-induced JNK, p38 and ERK1/2 activation.** Peritoneal macrophages were pretreated with CJ methanol extract 1 h followed by LPS stimulation for 15 min. Total protein was extracted and assessed by Western blotting. One of four experiments is shown. Data represent mean ± SD of four independent assays. * denotes significant difference ( *P* < 0.05) from cells treated with LPS alone.

We further investigated the effect of CJ methanol extract on the activations of JNK, p38, and ERK1/2, the major MAPKs that mediate LPS-induced signal transduction. Fifteen minutes of LPS activation sufficiently induced the phosphorylations of p38, JNK and ERK1/2, and treatment with CJ methanol extract caused inhibitions in all of these kinases (Figure 4). Among them, the effects on phospho-ERK and phospho-p38 were more prominent, indicating that different components of CJ may exert distinct roles.

### Effects of CJ methanol extract on STAT1 activation

**Figure 5:**
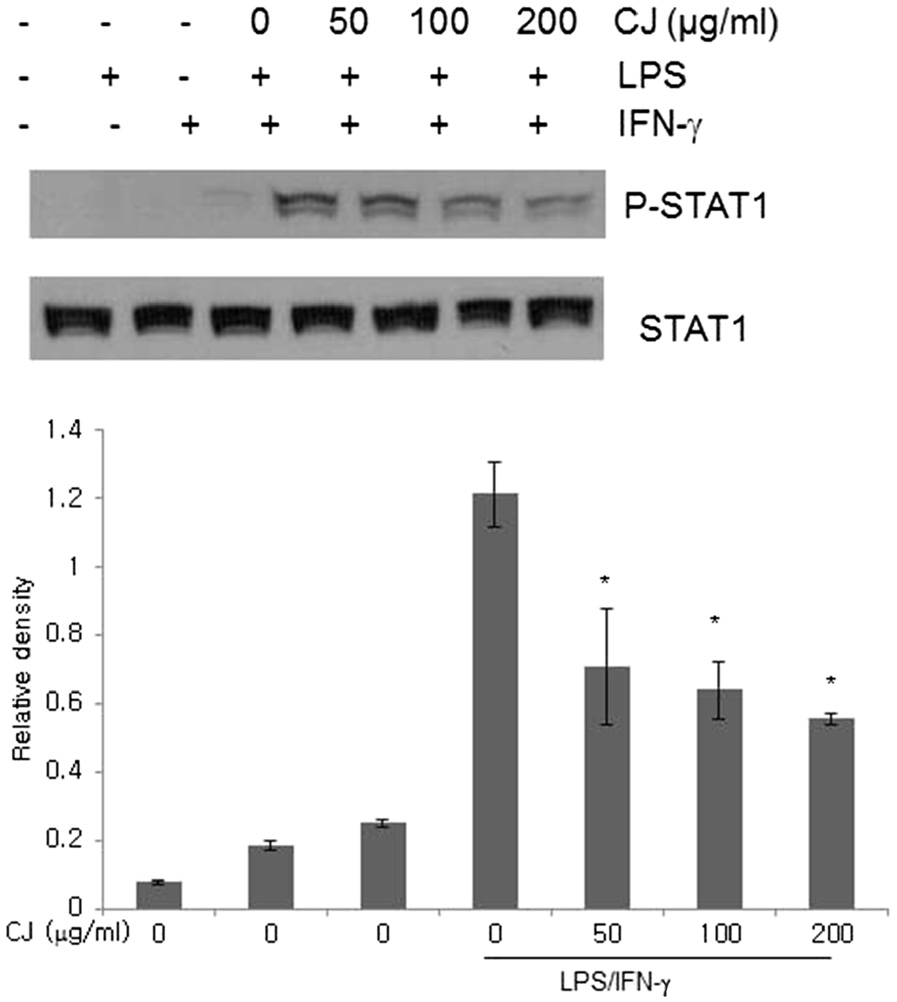
**Effect of CJ methanol extract on phospho-tyrosine of STAT1.** Macrophages were primed with IFN-γ for 2 h and treated with LPS and CJ methanol extract for 3 h. Total protein was extracted and assessed by Western blotting. One of four experiments is shown. Data represent mean ± SD of four independent assays. * denotes significant difference ( *P* < 0.05) from cells treated with LPS and IFN-γ.

STAT proteins are latent gene regulatory proteins that play an important role in cytokine-mediated intracellular signaling [9]. Binding of IFN-γ to its receptor causes STAT1 phosphorylation, which then translocates the proteins into the nucleus. We measured the levels of STAT1 phosphorylation at tyrosine 701. As shown in Figure 5, stimulation with either LPS or IFN-γ alone did not induce detectable STAT1 phosphorylation but LPS stimulation in IFN-γ-primed cells did. CJ methanol extract inhibited STAT1 activation in a concentration-dependent manner.

### In vivo effect of CJ methanol extract on the serum cytokines from LPS-injected mice

**Figure 6:**
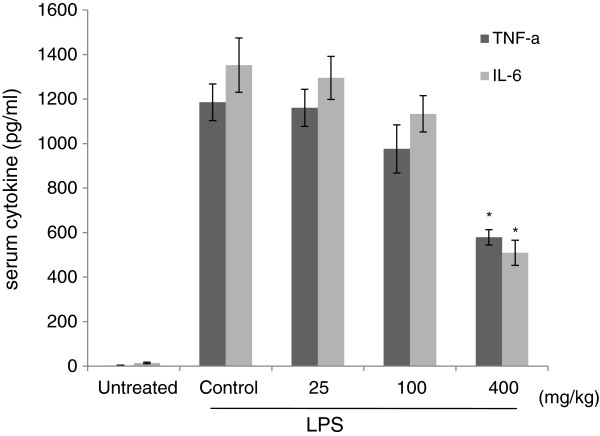
**Effect of CJ methanol extract on serum cytokines in LPS-injected mice.** CJ methanol extract ( 25, 100, and 400 mg/kg) was orally given to mice for 1 week. Untreated and control groups received an equal volume of water. On day 7 intraperitoneal injection of LPS (1.3 mg/kg) was carried out. Serum was obtained 1 h after LPS challenge. Data are mean ± SEM of three independent assays (n=13∼16). * : *P* <0.05 significant versus control group (ANOVA followed by Dunnet post hoc test).

Finally, we attempted to confirm the in vivo effect of CJ on acute inflammatory responses. Mice were given CJ for 1 week before intraperitoneal injection of LPS. CJ treated mice showed a dose-dependent decrease in serum TNF-α and IL-6 1 h after LPS challenge, but only the high dose group ( 400 mg/kg) reached statistical significance (Figure 6).

## Discussion

In this paper, we investigated the in vitro and in vivo anti-inflammatory effects of CJ methanol extract using the LPS-mediated model and found that the extract from this plant was able to suppress the productions of iNOS, TNF-α, IL-6, and IL-12 in activated macrophages. Also, CJ methanol extract inhibited LPS or LPS/IFN-γ-triggered intracellular signaling pathways that end in the activation of such molecules as IκBα, MAPK and STAT1.

NO is a signaling molecule; it diffuses into the cytosol of neighboring cells and binds to the iron cofactor of guanylate cyclase, triggering activation of the enzyme and elevating intracellular cGMP concentrations [10]. However, NO is also a free radical; it reacts with reactive oxygen species to produce peroxynitrite, a potent oxidant that inactivates target proteins by direct nitrosylation. The main control of NO production is determined by iNOS, and NF-κB, STAT1, and AP-1 are among the known transcription factors involved in the regulation of iNOS expression [11]. In particular, NF-κB is a target modulated by many iNOS inhibitors such as glucocorticoids and antioxidants [11]. IκBα degradation is critical for the regulation of NF-κB. IκBα is the prototypical protein of the IκB protein family (IκBα, IκBβ, IκBγ, IκBε, Bcl-3, p100, and p105) [8]. Phospho-IκBα is subject to polyubiquitination by E2 UbcH5 and E3 SCF^βTrCP^ and is then degraded by the 20S proteasome. Our results indicate that the action of CJ methanol extract occurred in the pathways linking LPS to IKK.

TNF-α and IL-6 play major roles in vascular permeability, neutrophil recruitment, blood clotting, and acute phase protein synthesis: all of which are characteristics of acute inflammation. IL-12 activates NK cells and promotes the differentiation of T-helper cells into IFN-γ-secreting Th1 cells, which enhance macrophage activity [12]. The MAPK signaling pathway mediates the LPS-triggered expressions of TNF-α, IL-6, and IL-12 [4,12]. The inhibitions of p38, JNK and ERK1/2 by CJ methanol extract may explain part of the mechanism that underlies the suppression of these pro-inflammatory cytokines.

IFN-γ upregulates the receptors for PAMP and DAMP, resulting in enhanced macrophage function. IFN-γ-dependent biological responses were impaired in STAT1-deficient mice [13]. STAT1 has two phosphorylation sites, one at tyrosine 701 and the other at serine 727 [14]. Phosphorylation at tyrosine 701 is a direct result of IFN-γ exposure while phosphorylation of serine 727 requires a separate signaling pathway. LPS is able to induce phosphorylation at tyrosine 701 in a delayed manner, but uses the same IFN-γ receptor-mediated pathway. Our experimental model utilized both IFN-γ and LPS to fully activate STAT1. Inhibition of STAT1 phosphorylation at tyrosine 701 by CJ methanol extract may contribute to the downregulation of macrophage activity.

Many medicinal and food plants that belong to the Apiaceae family contain bioactive polyacetylenes [15]. Falcarinol type polyacetylenes have been demonstrated to inhibit the release of NO and inflammatory cytokines in LPS-activated macrophages [16,17]. Catechol, a polyphenol found in CJ, has been reported to be a potent inhibitor of iNOS expression and NF-κB activation [18]. Presumably, part of the anti-inflammatory activity of CJ may be due to the presence of polyacetylene compound and catechol.

## Conclusions

Taken together, the aerial part of CJ methanol extract was effective in suppressing the production of iNOS, TNF-α, IL-6, and IL-12 in LPS-stimulated macrophages in vitro and in vivo. The anti-inflammatory action of this plant includes modulation of STAT1 and MAPK activation as well as IκBα degration. Future study is required to characterize the active compounds of CJ extract.

## Methods

### Sample preparation

The aerial parts of *Crytotaenia japonica* Hassk. were collected in the Medicinal Herb Garden of Kyung Hee Univeristy (Yongin) in May 2009. A voucher sample specimen (CJ-01) was deposited in the laboratory of Herbology, College of Oriental Medicine, Kyung Hee University. The dried plant was boiled three times in 100% methanol for 2 h. The extract was filtered, concentrated *in vacuo*, and dried with a lyophilizer. The yield of the extract was approximately 24.7%. The powdered extract was dissolved in dimethyl sulfoxide (DMSO) (Sigma, St. Louis, MO, USA) and sterilized by passing through a 0.22 μm syringe filter. A maximum of DMSO used for *in vitro* studies was 0.1%.

### Gas chromatography / mass spectrometery

One mg of CJ methanol extract dissolved in 0.01 ml of DMSO was examined by gas chromatography coupled with mass spectrometer (Perkin Elmer Clarus 600T). A DB-5MS capillary column (30m x 0.25mm, film thickness 0.25μm) was used for the separation of constituents. The column temperatures were programmed from 50°C hold in initial 3 min to 140°C hold in 8.5 min, and then 310°C hold in 35 min. A constant flow rate of 1.0 ml/min was applied by using helium as the carrier gas. The electron energy for the mass selective detector was 70 eV. The temperature of the ion source was set at 255°C. Mass selective detector was used in SCAN mode over a mass scan range at m/z 50 to 600.

### Animals

BALB/c mice (male, 8 weeks of age) were purchased from the Korean branch of Taconic, Samtaco (Osan, Korea), kept in a temperature-and humidity-controlled, pathogen-free animal facility at Kyung Hee University and provided standard mouse chow and water *ad libitum*. The mice were maintained in accordance with the Guide for the Care and Use of Laboratory Animals issued by the United States National Research Council (1996), and the protocol was approved by the Kyung Hee University Institutional Animal Care and Use Committee.

### Isolation of peritoneal macrophages

Mice were injected intraperitoneally with 2 ml of sterile thioglycollate medium (BD, Sparks, MD, USA). Three days later, macrophages were collected by peritoneal lavage with cold Dulbecco’s modified Eagle’s medium (DMEM). Cells were resuspended in DMEM with 10% fetal bovine serum and incubated for 2 h in a humidified atmosphere of 5% CO_2_ at 37°C. Non-adherent cells were removed and the resulting adherent cell population consisted of 95% macrophages, as determined by morphology and non-specific esterase staining.

### Viability assay

Cells were seeded at 4x10^4^/ 0.1 ml in 96-well plates and stimulated for 24 h at increasing concentrations of CJ methanol extract. Cell viability was determined using the 3-(4,5-dimethylthiazol-2-yl)-5-(3-carboxymethoxyphenyl)-2-(4-sulfopnehyl)-2H-tetrazolium (MTS) (Promega, Madison, WI, USA). Optical density was read at 490 nm with a microplate reader (Molecular Devices, Sunnyvale, CA, USA).

### Measurement of nitrites

Cells were seeded at 2x10^6^/ 2.0 ml in 6-well plates and primed for 2 h with 0.5 ng/ml of IFN-γ (BD Pharmingen, San Diego, CA, USA) before addition of LPS and CJ methanol extract. At 18 h after LPS stimulation, supernatant and cell pellets were used for subsequent assays. 50 μl medium was incubated with an equal volume of Griess reagent (Sigma) for 15 min at room temperature. The absorbance at 550 nm was measured with the microplate reader.

### Cytokine measurement

Supernatants or sera were appropriately diluted and the levels of cytokines were measured by ELISA according to the manufacturer’s protocol (BD Pharmingen).

### Analysis of signaling molecules

Cells were seeded at 3x10^6^/ 2.0 ml in 6-well plates and pre-treated for 1 h with CJ methanol extract and then stimulated with LPS for additional 15 min or 3 h. For the measurement of phospho-STAT1, cells were primed with IFN-γ.

### Western blotting

Total proteins were prepared by resuspending the cells in lysis buffer (50 mM Tris–HCl, pH 7.5; 150 mM NaCl; 1mM EDTA; 20mM NaF; 0.5% NP-40; and 1% Triton X-100) containing a phosphatase inhibitor (Sigma) and a protease inhibitor cocktail (Roche Diagnostics, Mannheim, Germany). Protein concentration was determined using the Bradford assay. Cell extracts were run on an 8% or 10% sodiumdodecyl sulfate-polyacrylamide gel and were transferred to polyvinylidene fluoride. The membranes were blocked with 5% skim milk in Tris-buffered saline with 0.1% Tween 20 (TBST) for 1 h and then incubated overnight at 4°C incubated with IκBα, β-tubulin (Santa Cruz Biotechnology, CA, USA), iNOS (BD Pharmingen), phospho-IκBα, phospho-JNK, JNK, phospho-p38, p38, phospho-ERK1/2, ERK1/2, phospho-STAT1, or STAT1 (Cell Signaling Technology, CA, USA) diluted 1/1000 in 5% skim milk in TBST. The blots were washed with TBST and incubated for 1 h with anti-rabbit or anti-mouse HRP-conjugated antibody (diluted 1:5000 in 5% skim milk in TBST). Immunoreactive bands were developed using an enhanced chemiluminescence system (GE Healthcare, Little Chalfont, Buckinghamshire, UK).

### In vivo experiment

CJ methanol extract dissolved in water ( 25, 100, and 400 mg/kg) was orally given for 1 week. On day 7, intraperitoneal injection of LPS (1.3 mg/kg) was performed and 1 h later mice were anesthetized with ether and blood was obtained by cardiac puncture.

### Statistical analysis

Statistical differences among the means of multiple groups were determined by using one way ANOVA followed by Dunnet’s post hoc test. The difference between the two means was assessed using non-paired student’s t test. Calculations were carried out using SPSS version 12. P values less than 0.05 were considered significant.

## Abbreviations

CJ: *Cryptotaenia japonica* Hassk; PAMP: Pathogen-associated molecular pattern; DAMP: Danger-associated molecular pattern; IFN-γ: Interferon-γ; NK cells: Natural killer cells; LPS: Lipopolysaccharide; NO: Nitric oxide; iNOS: Inducible nitric oxide synthase; TNF-α: Tumor necrosis factor-α; IL-6: Interleukin-6; IL-12: Interleukin-12; IκB: Inhibitor of κB; NF-κB: Nuclear factor-κB; MAPK: Mitogen-activated protein kinase; JAK: Janus kinase; STAT: Signal transducers and activators of transcription; ERK: Extracellular signal-related kinase; JNK: C-Jun N-terminal kinase; DMSO: Dimethyl sulfoxide; ELISA: Enzyme linked immunosorbent assay.

## Competing interests

The authors have no conflict of interest.

## Authors’ contributions

HK and HYC have written the paper. TSB, JWL, and JHL performed the in vitro experiments. KJL performed animal experiments. SHE performed chemical analysis. All authors have read and approved the final manuscript.

## Pre-publication history

The pre-publication history for this paper can be accessed here:

http://www.biomedcentral.com/1472-6882/12/199/prepub
